# Structural and functional analysis of the *Klebsiella pneumoniae* MazEF toxin–antitoxin system

**DOI:** 10.1107/S2052252521000452

**Published:** 2021-03-05

**Authors:** Chenglong Jin, Sung-Min Kang, Do-Hee Kim, Bong-Jin Lee

**Affiliations:** a The Research Institute of Pharmaceutical Sciences, College of Pharmacy, Seoul National University, Gwanak-gu, Seoul, 08826, Republic of Korea; b College of Pharmacy, Duksung Women’s University, Seoul, 01369, Republic of Korea; c College of Pharmacy, Jeju National University, Jeju, 63243, Republic of Korea; d Interdisciplinary Graduate Program in Advanced Convergence Technology & Science, Jeju National University, Jeju, 63243, Republic of Korea

**Keywords:** toxin–antitoxin systems, MazEF, *Klebsiella pneumoniae*, ribonuclease activity, structural homologs

## Abstract

Bacterial toxin–antitoxin (TA) systems correlate strongly with physiological processes in bacteria, such as growth arrest, survival and apoptosis. Here, the first crystal structure of a type II TA complex structure of *Klebsiella pneumoniae* at 2.3 Å resolution is presented. The *K. pneumoniae* MazEF complex consists of two MazEs and four MazFs in a heterohexameric assembly. It was estimated that MazEF forms a dodecamer with two heterohexameric MazEF complexes in solution, and a truncated complex exists in heterohexameric form. The MazE antitoxin interacts with the MazF toxin via two binding modes, namely, hydro­phobic and hydro­philic interactions. Compared with structural homologs, *K. pneumoniae* MazF shows distinct features in loops β1–β2, β3–β4 and β4–β5. It can be inferred that these three loops have the potential to represent the unique characteristics of MazF, especially various substrate recognition sites. In addition, *K. pneumoniae* MazF shows ribonuclease activity and the catalytic core of MazF lies in an RNA-binding pocket. Mutation experiments and cell-growth assays confirm Arg28 and Thr51 as critical residues for MazF ribonuclease activity. The findings shown here may contribute to the understanding of the bacterial MazEF TA system and the exploration of antimicrobial candidates to treat drug-resistant *K. pneumoniae*.

## Abstract 2

The first crystal structure of a type II toxin–antitoxin complex structure of *Klebsiella pneumoniae* at 2.3 Å resolution is presented. Mutational experiments and cell-growth assays confirm Arg28 and Thr51 as critical residues for MazF ribonuclease activity.

## 1. Introduction   

Toxin–antitoxin (TA) systems were originally discovered as plasmid maintenance systems in almost all free-living bacteria, in which only daughter cells harboring the TA operon can survive (Yamaguchi *et al.*, 2011 ▸). TA systems are strongly correlated with physiological processes such as gene regulation, growth arrest, survival and apoptosis (Goeders & Van Melderen, 2014 ▸). TA systems are typically encoded in operons consisting of adjacent toxin and antitoxin genes, and transferred to daughter cells to yield plasmid stabilization and cell viability (Fernández-García *et al.*, 2016 ▸). In the normal state, the transcription of the toxin gene is coupled with that of its cognate antitoxin gene, and the antitoxin blocks the toxicity of the toxin (Lobato-Márquez *et al.*, 2016 ▸). However, unfavorable circumstances, such as nutrient deficiency, antibiotic treatment, environmental stress, plasmid loss, bacteriophage infection, immune system attack, oxidative stress and high temperature, induce a decrease in antitoxin concentration, leading to increased levels of free toxin and in turn to growth arrest and eventually cell death (Kang *et al.*, 2018 ▸).

TA systems can be typically classified into six different types according to the mechanism by which the toxin is neutral­ized by the antitoxin. In type I TA systems, the antitoxin is an antisense RNA that forms base pairs with toxin mRNA and thereby inhibits toxin synthesis (Soutourina, 2019 ▸). In type II TA systems, both the toxin and the antitoxin are small proteins. Under normal conditions, the labile antitoxin interacts with the toxin, resulting in neutralization of the toxin and the forming of a nontoxic complex (Rocker & Meinhart, 2016 ▸). In type III TA systems, the antitoxin is an RNA that makes a specific interaction with cognate toxin protein to form the RNA pseudoknot–toxin complex, resulting in its neutral­ization (Goeders *et al.*, 2016 ▸). In type IV TA systems, the antitoxin and the toxin do not interact directly but participate in cytoskeleton assembly by competing for the same proteins, such as MreB and FtsZ (Jankevicius *et al.*, 2016 ▸). In type V TA systems, the antitoxin acts as a ribonuclease that specifically degrades its cognate toxin mRNA to prevent its expression. In type VI systems, both the toxin and the antitoxin are proteins (Wang *et al.*, 2013 ▸). Under normal conditions, the toxin is delivered by the antitoxin to a cellular protease, resulting in its degradation and promoting DNA replication (Aakre *et al.*, 2013 ▸).

*K. pneumoniae* strain ATCC 700721 contains nine type II TA systems. In a type II TA system, the toxin is thermodynamically stable; in contrast, the antitoxin is unstable and rapidly degraded by proteases of the Clp or Lon family because its locally flexible conformation makes it susceptible to proteolysis. Once degradation of the antitoxin occurs, the free toxin is released from the nontoxic complex, causing growth arrest or even death of the host cell (Syed & Lévesque, 2012 ▸). MazEF is classified as a type II TA system and has been strongly implicated in programmed cell death (Ramisetty *et al.*, 2015 ▸). When activated, MazF cleaves RNA, and subsequent processes result in cell death (Engelberg-Kulka *et al.*, 2006 ▸). Therefore, it is hypothesized that the MazEF system closely mediates genetic programs that promote bacterial cell death (Lee & Lee, 2019 ▸).

Here, we present the crystal structure of *K. pneumoniae* MazEF at 2.3 Å resolution, which is the first TA complex structure of *K. pneumoniae*. The structure clearly shows the binding interface between MazE and MazF and the active site responsible for ribonuclease activity. Mutational experiments and cell-growth assays confirm Arg28 and Thr51 as critical residues for MazF ribonuclease activity. Our findings will contribute to the understanding of the bacterial MazEF TA system.

## 2. Materials and methods   

### 2.1. Cloning and transformation   

The genes encoding *K. pneumoniae* MazE (*kpn_pkpn7p10263*) and MazF (*kpn_pkpn7p10264*) were amplified using polymerase chain reaction (PCR). Two kinds of MazE antitoxins were used in the following experiments. To obtain high-resolution diffracted crystals, MazE truncated from Lys45 to Leu76 was used for crystallography. Thus, the primers used for PCR were as follows: truncated MazE, forward, 5′-G GAA TTC CAT ATG AAA GCT GGC CCG ACG C-3′, and reverse, 5′-CCG CTC GAG TTA CAG CAT CTC CTT ACC-3′; MazF, forward, 5′-G GAA TTC CAT ATG ACG ACA TAT TGT CCA G-3′, and reverse, 5′-CCG CTC GAG TTA TGC TTT AAT AAT TTT TG-3′. Nde1 and Xho1 were used as restriction enzymes for cloning, and the restriction sites are underlined above. The PCR products of MazE and MazF were doubly cleaved by Nde1 and Xho1 and ligated into vectors that had been cleaved by the same enzymes. For structure determination, MazE and MazF were ligated into pET28a and pET21a with no tags, respectively. For the biological assay, MazF was ligated into pET28a with an additional residual tag whose amino-acid sequence was MGSSHHHHHHSSGLVPRGSH. Each plasmid was then transformed into *Escherichia coli* DH5α competent cells.

### 2.2. Protein expression and purification   

For the structure determination of the *K. pneumoniae* MazEF complex, plasmids containing the MazE antitoxin and MazF toxin genes were cotransformed into *E. coli* Rosetta2 (DE3) pLysS competent cells. The cells were grown in Luria broth at 37°C until the OD_600_ of the cell culture reached 0.6. Iso­propyl 1-thio-β-d-galactopyran­oside (IPTG, 0.5 m*M*) was added for target-protein overexpression, and the cells were further incubated at 37°C for 4 h. The cultured cells were harvested by centrifugation at 11 355*g* at 10°C and stored in a −82°C deep freezer. The harvested cells were then suspended in buffer *A* (20 m*M* Tris–HCl, pH 7.9, 500 m*M* NaCl) containing 10%(*v*/*v*) glycerol and lysed by ultrasonication. After centrifugation at 28 306*g* for 1 h, the supernatant containing soluble proteins was filtered using a 0.45 µ*M* syringe filter (Sartorius) and loaded onto an affinity chromatography column of nickel-nitrilo­tri­acetic acid-agarose (Bio-Rad) that had been equilibrated with buffer *A*. The column was washed with buffer *A* containing 50 m*M* imidazole, and the target protein MazEF bound to Ni^2+^ resin was eluted using an imidazole gradient (100–700 m*M*). For further purification of MazEF using ion-exchange chromatography, the elution buffer was exchanged with buffer *B* (20 m*M* Tris, pH 8.0), and the eluted protein was loaded onto a HiTrap Q column (GE Healthcare) pre-equilibrated with the same buffer. The protein was then eluted with a NaCl gradient (0–1000 m*M*). As the final purification step, size-exclusion chromatography (SEC) on a HiLoad 16/600 Superdex 200 prep-grade column was used for buffer exchange to final buffer (20 m*M* Tris, pH 8.0, 50 m*M* NaCl) and for conducting the final MazEF purification. In the above experimental steps, the target protein was identified using SDS–PAGE and concentrated using an Amicon Ultra centrifugal filter unit (Millipore). Finally, 7.5 mg ml^−1^ MazEF was used for crystallization.

For the MazF ribonuclease-activity assay, the MazF toxin was overexpressed in *E. coli* Rosetta2 (DE3) pLysS competent cells. However, the cells were incubated for only 1 h after IPTG induction because of the toxicity of MazF to *E. coli*. The purification procedures were the same as for the MazEF complex, except that ion-exchange chromatography was omitted for MazF toxin since no contamination appeared in the purification of MazEF. The final buffer containing MazF was 20 m*M* Tris, pH 8.0, 200 m*M* NaCl.

### 2.3. Crystallization, data collection and processing   

Initial crystal screening of the MazEF complex was conducted using the Wizard classic 1, 2 and 3, 4 (Rigaku) and Index 1 and 2 (Hampton Research) kits, by mixing 0.5 µl of protein solution at 7.5 mg ml^−1^ in 20 m*M* Tris, pH 8.0, 50 m*M* NaCl with 0.5 µl of reservoir solution. Crystals of the MazEF complex were grown using the sitting-drop vapor-diffusion method at 20°C. The crystallization solution for the MazEF complex was 5%(*w*/*v*) PEG 1000, 100 m*M* sodium phosphate dibasic/citric acid at pH 4.2 and 40%(*v*/*v*) reagent alcohol. The crystal was transferred to cryoprotectant solution containing 20%(*v*/*v*) glycerol in the reservoir prior to mounting. Furthermore, the crystals were cooled using liquid nitro­gen immediately prior to data collection. The diffraction data were collected using the ADSC Quantum Q270r CCD detector at the 5C and 11C beamlines of Pohang Light Source, Republic of Korea. The crystals of the MazEF complex belong to the triclinic *P*1 space group, with unit-cell parameters of *a* = 41.558, *b* = 45.501, *c* = 69.952 Å, and α = 102.06, β = 93.60, γ = 116.36°. The calculated total mass of the MazEF complex, including the N-terminal (His_6_) tag, was 29.8 kDa. All raw data were scaled and processed using *HKL2000* (Otwinowski & Minor, 1997 ▸). As a result, the structure of the MazEF crystal was initially phased by molecular replacement using *Phenix* (Liebschner *et al.*, 2019 ▸), as based on the crystal structure of *E. coli* MazF toxin [PDB code 1ub4 (chain A); Kamada *et al.*, 2003 ▸] as a template. Then, the structure of MazEF was autobuilt using its own sequence file and diffraction data of 2.3 Å resolution. We used a criteria of CC_1/2_ > 0.9 for the resolution cutoff. Therefore, the resolution of data used in this study is 2.3 Å. However, since several residues from the MazE antitoxin were not correctly matched in the electron-density map, the subsequent manual model building of MazEF was conducted using *Coot* (Emsley *et al.*, 2010 ▸). Furthermore, the models were recurrently refined using *REFMAC5* (Murshudov *et al.*, 2011 ▸) and *Phenix* (Liebschner *et al.*, 2019 ▸). The detailed crystallographic statistics are summarized in Table 1 ▸. The overall geometry of MazEF was validated using *MolProbity* (Chen *et al.*, 2010 ▸). The electrostatic potential surfaces were calculated using the adaptive Poisson–Boltzmann solver method (Baker *et al.*, 2001 ▸). *PyMOL* (Schrödinger, LLC, USA) was used to generate all of the structural figures (Delano, 2002 ▸).

### 2.4. *In vitro* ribonuclease assay   

To validate the ribonuclease activity of MazF toxin, an RNase alert kit (IDT) was used. The principle is as follows. In the normal state, a fluoro­phore is covalently attached to one end of a synthetic RNA strand and is quenched by a quencher group at the other end of the RNA strand. However, the synthetic RNA is digested and the quencher is released when the ribonuclease interacts with substrates. Then, the released fluoro­phore emits fluorescence at 520 nm upon excitation at 490 nm. The resulting relative fluorescence units (RFU) were detected using a SPECTRAmax GEMINI XS spectro­fluoro­meter (Molecular Devices). We first investigated the concentration-dependent ribonuclease activity of MazF, whose concentration was gradually increased with values of 0.5, 1, 2, 4 and 10 µ*M*. Metal-dependent ribonuclease activity was also investigated using different kinds of metal ions, such as Mg^2+^, Ca^2+^, Mn^2+^ and Zn^2+^. The concentrations of MazF toxin and metal ions were fixed at 4 and 50 µ*M*, respectively. All experiments were performed in triplicate.

### 2.5. Mutational study on the active site   

For the mutational study of the key catalytic residues of MazF, Arg28, Thr51, Lys79 and Arg86 were mutated to alanine. The resulting mutant proteins were designated R28A, T51A, K79A and R86A. Mutation was conducted using the EZchange site-directed mutagenesis kit (Enzynomics, Republic of Korea) according to the manufacturer’s protocol. Mutated MazF proteins were expressed and purified using the same procedures as were used for the native proteins.

### 2.6. Cell-growth assay   

For the cell-growth assay, the plasmids expressing MazF, R28A, T51A, K79A and R86A, were transformed into *E. coli* strain Rosetta (DE3) pLysS. Transformed cells from single colonies grown on 0.1% glucose-containing M9 medium plates were grown overnight, and the overnight cultures were diluted to an OD_600_ of 0.1. The diluted cells were further grown until the OD_600_ of the cell suspension reached 0.3, at which time 0.5 m*M* IPTG was added to induce protein expression. The cells were incubated at 37°C for 6 h after induction by IPTG and monitored at 1 h intervals.

## 3. Results and discussion   

### 3.1. Overall structure of the MazEF complex   

**Figure 1:**
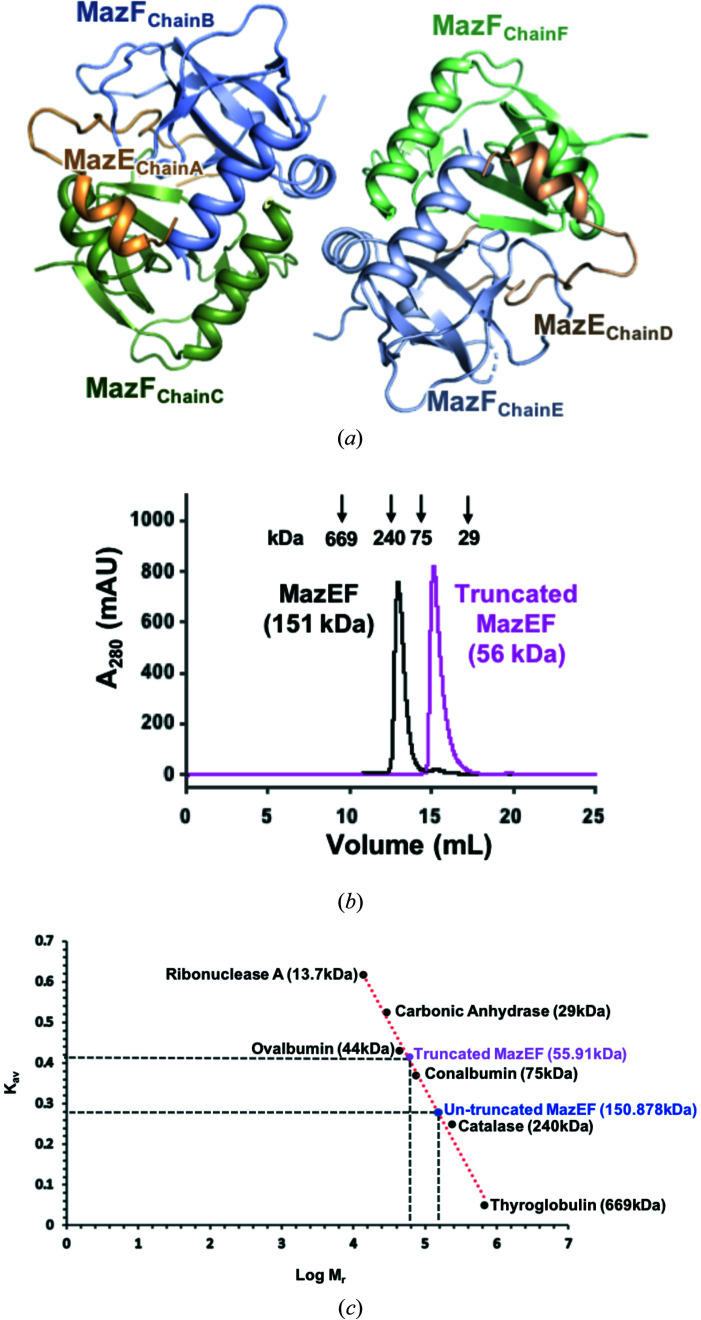
The overall structure and oligomeric state of MazEF (*a*) A cartoon representation of the MazEF heterohexamer. Chains A and D of the MazE antitoxin are shown in orange. Chains B, C, E and F of the MazF toxin are shown in light blue and green. (*b*) Superposed SEC chromatograms of full-length and truncated MazEF. (*c*) The standard curve of a HiLoad 16/600 Superdex 200 prep-grade column is shown as a red dotted line. The elution volumes of truncated and untruncated MazEF projected on this line allow the determination of the corresponding molecular masses.

The crystal structure of the MazEF complex from *K. pneumoniae* was determined at a resolution of 2.3 Å. The asymmetric unit of the MazEF complex is composed of two MazE antitoxins and four MazF toxins in a heterohexameric assembly [Fig. 1 ▸(*a*)]. By comparison with reference proteins in gel-filtration calibration kits (GE Healthcare) using SEC, the predicted molecular weights of both truncated and untruncated MazEF complexes in solution were obtained. In SEC, untruncated MazEF elutes at ∼12.97 ml, while truncated MazEF elutes at ∼15.11 ml [Fig. 1 ▸(*b*)]. These values correspond to molecular masses of 150.878 and 55.91 kDa, respectively, using the calibration curve for several standard proteins [Fig. 1 ▸(*c*)]. Because the theoretical molecular weights of the untruncated and truncated heterohexameric MazEF complexes are 73.9 and 59.5 kDa, respectively, it can be estimated that MazEF forms a dodecamer with two heterohexameric MazEF complexes in solution, and the truncated complex exists in heterohexameric form.

**Figure 2:**
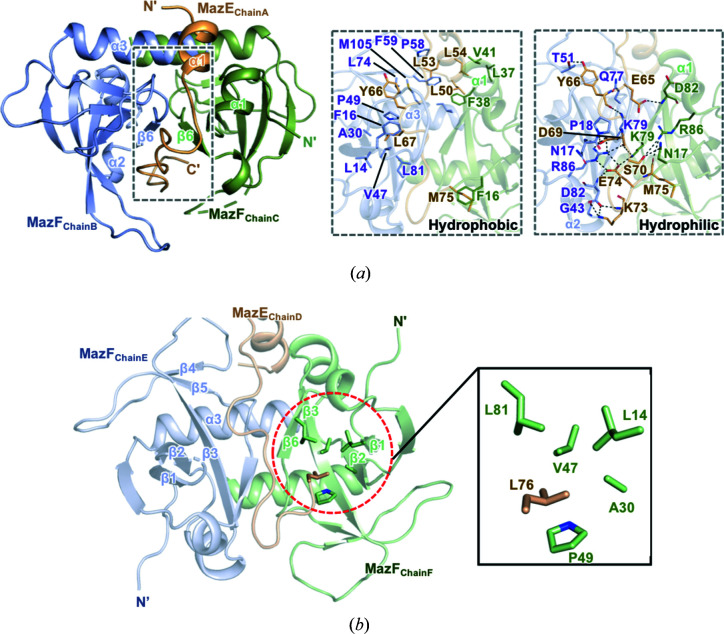
The heterotrimeric interface of MazE and MazF. (*a*) Details of the heterotrimeric interface between the MazE and MazF dimers. Residues participating in hydro­phobic interactions and hydro­philic interactions are shown as stick models. Hydrogen bonds are shown as black dotted lines. (*b*) The hydro­phobic core formed around Leu76 (chain D). All of the residues participating in the hydro­phobic core are shown in stick representation and labeled.

In the heterotrimeric subunit of MazEF, the MazE monomer was positioned between symmetrically arrayed MazF dimers [Fig. 2 ▸(*a*)]. The MazE monomer interacts with the MazF homodimer via its long C-terminus loop, which wraps around the MazF dimer and extends further towards the empty edge between MazF monomers.

The MazE antitoxin interacts with the MazF toxin via two binding modes, namely, hydro­phobic and hydro­philic interactions. The calculation of surface area and analysis of interactions between MazE and MazF were conducted using *PISA* (Krissinel & Henrick, 2007 ▸) and the *PIC* server (Tina *et al.*, 2007 ▸). The long C-terminus loop of MazE contributes to binding with MazF through hydro­phobic interactions involving the following MazE residues: Leu50, Leu53, Leu54, Tyr66, Leu67 and Met75 (chain A); and the following MazF residues: Leu14, Phe16, Ala30, Val47, Pro49, Pro58, Pro59, Leu74, Leu81, Met105 and Ile109 (chain B), and Phe16, Leu37, Phe38 and Val41 (chain C) [Fig. 2 ▸(*a*)]. Furthermore, the residues Leu14, Ala30, Val47, Pro49 and Leu81 in MazF (chain F) make hydro­phobic interactions with Leu76 (chain D) in MazE by forming a hydro­phobic core, which could only be observed between chain D and chain F owing to the lack of electron density for Leu76 in chain A [Fig. 2 ▸(*b*)].

Additionally, the C terminus of MazE contributes to the hydro­philic interaction between MazE and MazF, with several residues involved in hydrogen bonding or salt bridges. In detail, the following residues are necessary for forming hydrogen bonds: Glu65, Tyr66, Asp69, Ser70, Lys73, Glu74 and Met75 of MazE (chain A); Asn17, Pro18, Gly43, Thr51, Gln77, Lys79, Asp82 and Arg86 of MazF (chain B); and Asn17, Lys79, Asp82 and Arg86 of MazF (chain C) [Fig. 2 ▸(*a*)]. In addition, 13 salt bridges are formed between MazE (chain A) and MazF (chain B) and three salt bridges are formed between MazE (chain A) and MazF (chain C). Furthermore, the interface area of chain A and chain B is 1123.8 Å^2^ and the interface area of chain A and chain C is only 549.8 Å^2^. Therefore, it can be inferred that chain A and chain B have stronger binding capacity than chain A and chain C.

### 3.2. Overall structure of the MazF homodimer   

**Figure 3:**
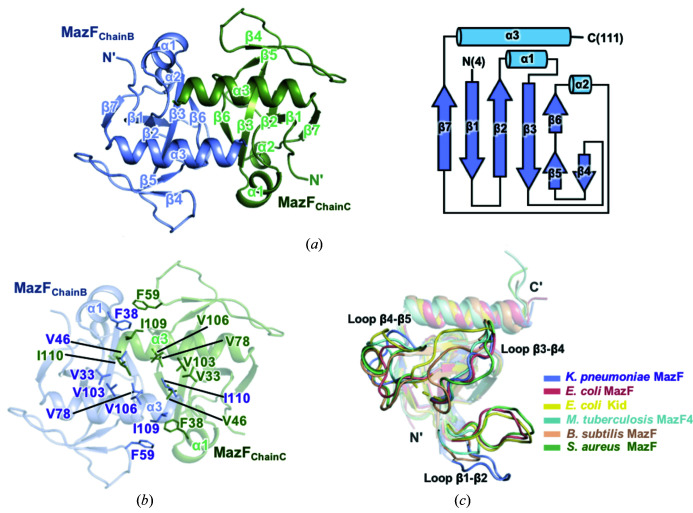
Structural features of the MazF toxin. (*a*) MazF consists of seven antiparallel β-strands flanked by three α-helices. A cartoon representation of the MazF dimer (left). A topology diagram with secondary structural elements of MazF (right). (*b*) The residues involved in hydro­phobic interactions are shown in stick representation. A hydro­phobic core is formed around the α3-helices. (*c*) Superimposition of the MazF monomer with its structural homologs. Five homologs were used for comparison. The conformations of loops β1–β2, β3–β4 and β4–β5 are quite different from each other.

The structure of *K. pneumoniae* MazF includes seven antiparallel β-strands flanked by three α-helices. The architecture of MazF adopts a β-barrel-like structure in the following order: β1 (residues 10–16), β2 (residues 26–32), α1 (residues 36–42), β3 (residues 44–51), β4 (residues 60–62), β5 (residues 70–74), α2 (residues 75–77), β6 (residues 79–82), β7 (residues 89–93) and α3 (residues 96–109) [Fig. 3 ▸(*a*)].

Overall, more than 15 residues are involved in the dimerization of MazF. The homodimeric interface of MazF buries an area of 911 Å^2^. Although hydrogen bonds produce a certain influence on the formation of the dimeric interface, the main driving forces of MazF dimerization are hydro­phobic interactions. In detail, a hydro­phobic core is formed around the α3-helices, which are located facing each other. In this hydro­phobic core, the α3-helix of one MazF monomer contacts the α3-helix, α1-helix and β3-strand of the other proximal monomer. Nine residues are involved in this interaction between two MazF monomers, among which Val46 and Ile110 are the key residues necessary for the dimerization of MazF. Ile110 in the α3-helix is involved in hydro­phobic interactions with Val33, Val46, Val103 and Val106. In addition, Ile46 in the β3-strand participates in hydro­phobic interactions with Val78, Ile109 and Ile110 [Fig. 3 ▸(*b*)].

To compare the tertiary structures of MazF toxin and its homologs, the *DALI* (Holm & Rosenstrom, 2010 ▸) server was primarily used to search for structural homologs of *K. pneumoniae* MazF. Several structural homologs were found, and the structures of the five closest homologs are: the MazF toxin from *E. coli* [PDB code 5cr2 (chain A) (Zorzini *et al.*, 2016 ▸), with an r.m.s. deviation of 1.8 Å, a *Z* score of 17.1 and a sequence identity of 44%], the Kid toxin from *E. coli* [PDB code 1m1f (chain A) (Hargreaves *et al.*, 2002 ▸), with an r.m.s. deviation of 2.5 Å, a *Z* score of 15.6 and a sequence identity of 35%], the MazF4 toxin from *Mycobacterium tuberculosis* [PDB code 5xe3 (chain A) (Ahn *et al.*, 2017 ▸), with an r.m.s. deviation of 2.0 Å, a *Z* score of 13.9 and a sequence identity of 23%], the MazF toxin from *Bacillus subtilis* [PDB code 4me7 (chain B) (Simanshu *et al.*, 2013 ▸), with an r.m.s. deviation of 2.2 Å, a *Z* score of 13.3 and a sequence identity of 30%] and the MazF toxin from *Staphylococcus aureus* [PDB code 5dlo (chain A) (Zorzini & Loris, unpublished work), with an r.m.s. deviation of 2.5 Å, a *Z* score of 12.9 and a sequence identity of 28%].

Interestingly, although they are from different kinds of bacterial strains, MazF toxin shows high *Z* scores with its structural homologs. The structural-alignment results show that the tertiary structures of the homologs approximately overlap, with the exception of loops β1–β2, β3–β4 and β4–β5 [Fig. 3 ▸(*c*)]. Thus, it can be inferred that these three loops have the potential to represent unique characteristics of MazF, especially various substrate recognition sites.

**Figure 4:**
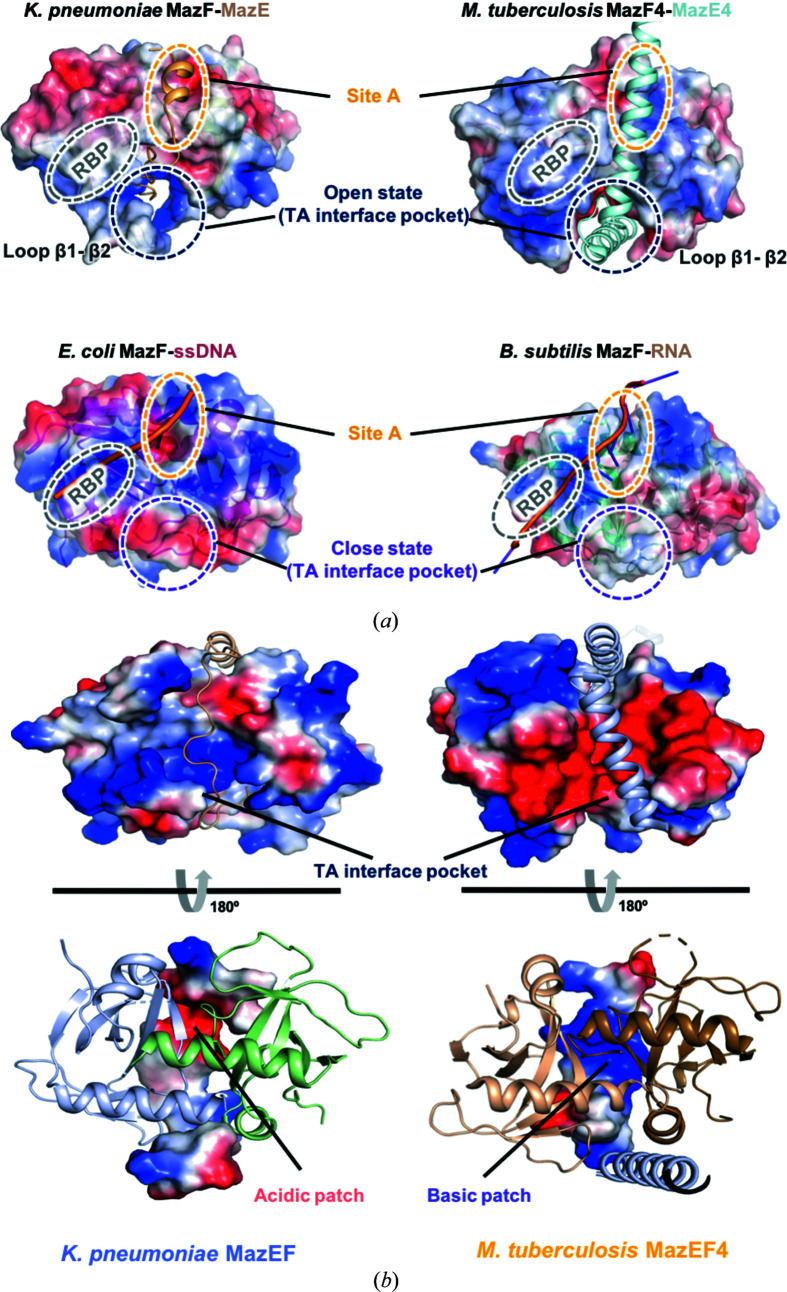
A structural study based on electronic surface potential. (*a*) Comparison of antitoxin-bound MazF with substrate-bound MazF via electronic surface potential. MazF-dimer transition between the open and closed states in the TA interface pocket through conformational change of loop β1–β2. Site A (yellow oval): the specific binding pocket of the downstream region of the RNA substrate. RBP (gray oval): the specific binding pocket of the upstream region of the RNA substrate. (*b*) Opposite electrostatic surface potential in TA interface pockets between *K. pneumoniae* MazEF and *M. tuberculosis* MazEF4. The electrostatic surface potential of MazF toxin along with cartoon representations of *K. pneumoniae* MazE (wheat) and *M. tuberculosis* MazE (light blue) (upper). The electrostatic surface potential of the C terminus of *K. pneumoniae* MazE and *M. tuberculosis* MazE4 along with cartoon representations of *K. pneumoniae* MazF (chain E: blue, chain F: green) and *M. tuberculosis* MazF (chain A: orange, chain B: brown) (lower). The TA interface pocket and charged MazF-binding patches are marked.

Among them, loop β1–β2 plays the most important role, as it is long enough to be involved in RNA recognition and functions as a gate to modulate an ‘open’ to ‘closed’ state (Hoffer *et al.*, 2017 ▸) [Fig. 4 ▸(*a*)]. In the closed state, loop β1–β2 extends to the adjacent toxin monomer to conceal the adjacent region where the MazE antitoxin binds. In contrast, binding of the MazE antitoxin not only displaces the β1–β2 linker to induce an open state but also occupies ‘Site A’ of the MazF homodimer. As a result, the interactions between MazF monomers are disrupted, especially in the ‘TA interface pocket’ (Simanshu *et al.*, 2013 ▸; Zorzini *et al.*, 2016 ▸). In addition, although a positively charged RNA-binding pocket (RBP) still exists in the open state, the substrate can no longer bind to MazF because Site A is regarded as the specific binding pocket of the downstream region of the substrate (Zorzini *et al.*, 2016 ▸). Notably, in the *K. pneumoniae* MazEF TA interface pocket, the major MazE-binding region is highly positively charged, which is opposite to its structural homolog *M. tuberculosis* MazF4 (PDB code 5xe3) (Ahn *et al.*, 2017 ▸) [Fig. 4 ▸(*b*)]. Thus, the basic principles by which these structural homologs recognize specific RNA via loop β1–β2 are somewhat different. In detail, when MazF homologs with a long loop β1–β2, such as *K. pneumoniae* MazF, *E. coli* MazF and *B. subtilis* MazF in ‘closed’ states, the TA interface pocket is concealed owing to the extended interactions of the relatively long loop β1–β2, which was mentioned above. As a result, the RBP exists only on the left ‘hemisphere’ of the MazF homodimer [Fig. 4 ▸(*a*)]. In contrast, loop β1–β2 of *M. tuberculosis* MazF4 is too short to close the corresponding pocket completely and hence exposes negatively charged binding pockets (Ahn *et al.*, 2017 ▸). However, substrates could not bind in this pocket owing to the repulsive forces between the same charge. Thus, it is suggested that in this case an RBP also exists in the same location as in the other structural homologs of MazF (Ahn *et al.*, 2017 ▸). In addition, it can be inferred that the catalytic core of MazF lies in this RBP.

### 3.3. Catalytic core of MazF   

**Figure 5:**
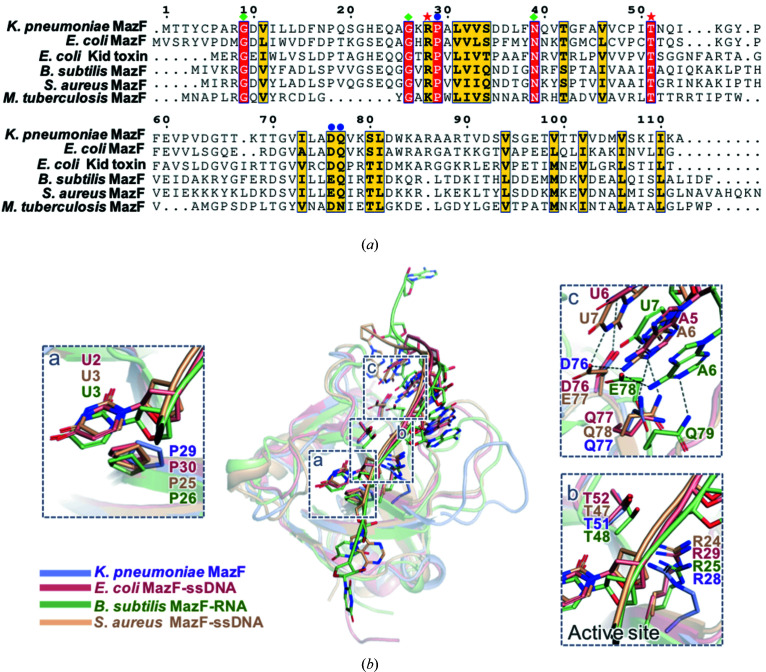
Sequence alignment showing several highly conserved residues in the MazF toxin. (*a*) Sequence alignment of *K. pneumoniae* MazF and five structural homologs. Identical and similar residues are highlighted in red and yellow, respectively. The conserved residues involved in the formation of the tertiary structure of MazF, interaction with the substrate and RNA catalysis are indicated by green squares, blue circles and red pentagrams, respectively. (*b*) Superimposition of the MazF monomer with its substrate-bound structural homologs. Three substrate-bound MazF toxins were used for comparison. *a*: Proline undergoing stacking interactions with the uracil base of the substrate. *b*: Arginine and threonine are highly conserved in structural homologs of MazF. *c*: Aspartate (glutamate) and glutamine tightly interact with adenosine and uracil via hydrogen bonding.

To identify the active sites of *K. pneumoniae* MazF, sequence alignment was conducted using *Clustal Omega* 1.2.1 (McWilliam *et al.*, 2013 ▸) and visualized using *ESPript* 3.0 (Robert & Gouet, 2014 ▸) [Fig. 5 ▸(*a*)]. The sequence of *K. pneumoniae* MazF was aligned with those of five structural homologs from different types of bacterial strains, which were mentioned in the previous subsection. The results revealed several highly conserved residues: Gly9, Gly26, Arg28, Pro29, Asn39, Thr51, Asp76 and Gln77. These residues can be divided into three categories via their mechanism of action in MazF: (i) Gly9, Gly26 and Asn39; (ii) Pro29, Asp76 and Gln77; and (iii) Arg28 and Thr51. In detail, residues in category (i) are related to the formation of the tertiary structure of MazF, while those in categories (ii) and (iii) are involved in interacting with RNA substrates. For example, the roles of glycines correspond to hydrogen-bonded turns of the loops and asparagines improve the stability of α1-helices. Since the *K. pneumoniae* MazEF crystal structure was not determined with the RNA substrate bound, it was easier to illustrate the roles of the residues in categories (ii) and (iii) by superimposing *K. pneumoniae* MazF with the RNA substrate-bound structures of *E. coli* MazF (PDB code 5cr2) (Zorzini *et al.*, 2016 ▸), *B. subtilis* MazF (PDB code 4mdx) (Simanshu *et al.*, 2013 ▸) and *S. aureus* MazF (PDB code 5dlo) (Zorzini & Loris, unpublished work) [Fig. 5 ▸(*b*)]. Although different constructs of RNA substrates were used for crystallization, each of them contained the common RNA sequence (UACAU). Among them, Pro29 makes hydro­phobic contact with the uracil base (U^1^) of the substrate through interaction with its side chain. Therefore, it is the most important residue defining substrate specificity (Zorzini *et al.*, 2014 ▸). Asp76 and Gln77 are also key residues involved in substrate recognition, which tightly interact with the downstream region (A^4^U^5^) of the specific RNA sequence via hydro­philic interactions. A previous study reported that MazF became inactive *in vivo* when aspartate and glutamine were mutated to alanine. Additionally, isothermal-titration-calorimetry results revealed that these mutations resulted in weaker RNA-binding affinity compared with wild-type (WT) protein (Simanshu *et al.*, 2013 ▸).

Furthermore, Arg28 and Thr51 are highly conserved in several structural homologs and are located on either side of the substrate in the RBP [Fig. 5 ▸(*b*)].

### 3.4. *In vitro* ribonuclease activity of MazF   

**Figure 6:**
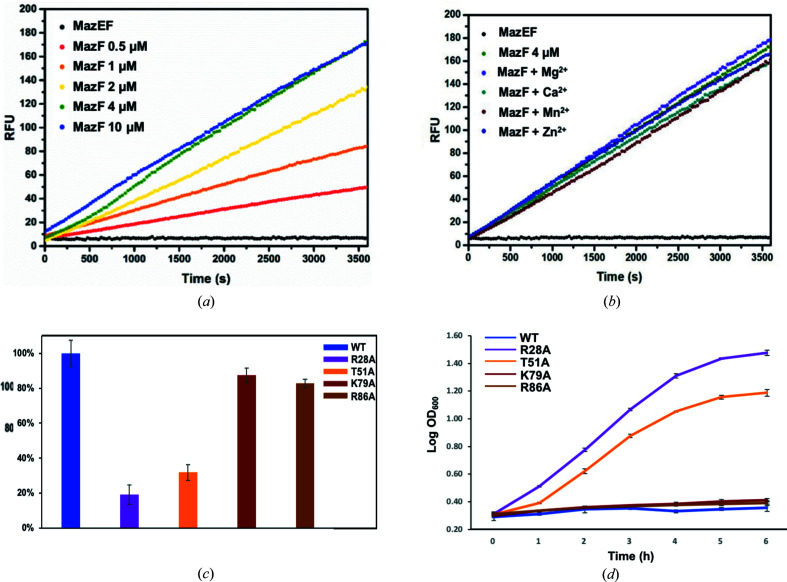
*In vitro* ribonuclease-activity assay. (*a*) Fluorescence measurements as a function of time during the addition of increasing amounts of MazF. Various concentrations of MazF monomer were prepared; at concentrations greater than 4 µ*M*, the reaction was saturated. (*b*) A metal-dependent ribonuclease-activity assay was conducted using several divalent metal ions. Notable changes were not observed in this assay. (*c*) An *in vitro* ribonuclease assay of *K. pneumoniae* MazF. The RFU obtained with 4 µ*M* WT MazF was taken as 100%. (*d*) An *in vivo* cell-growth assay of *K. pneumoniae* MazF. The growth of *E. coli* Rosetta (DE3) pLysS was monitored over 6 h after induction of WT MazF and several mutants of MazF. The error bars represent the standard error of the means from three independent experiments.

To determine the concentration of MazF toxin used for several assays in the following subsections, the ribonuclease activity of *K. pneumoniae* MazF was confirmed by the increase in the resulting fluorescence (RFU) with increasing concentration of the MazF monomer protein from 0.5 to 10 µ*M*. The reaction was saturated at concentrations greater than 4 µ*M* [Fig. 6 ▸(*a*)]; thus, the concentration of MazF was fixed at 4 µ*M* in all of the following RNase-activity assays. Furthermore, different kinds of divalent metal ions had no great effect on the RNase activity of MazF [Fig. 6 ▸(*b*)]. This means that the catalytic process of MazF is independent of metal ions, whereas the catalytic processes of other ribonuclease proteins, such as VapC 26 and VapC 30, in *M. tuberculosis* are highly mediated by divalent metal cations (Lee *et al.*, 2015 ▸; Kang *et al.*, 2017 ▸).

To demonstrate that Arg28 and Thr51 play a critical role in *K. pneumoniae* MazF ribonuclease activity, several mutants (R28A, T51A, K79A and R86A) were designed. Among them, Lys79 and Arg86, which are far from the active site, are only involved in hydro­philic interaction between MazE and MazF. As a result, two mutants of *K. pneumoniae* MazF (R28A and T51A) showed a significant reduction in ribonuclease activity *in vitro* compared with mutants (K79A and R86A) of MazF and WT MazF [Fig. 6 ▸(*c*)]. In addition, the results of the bacterial-growth assay revealed that expression of R28A and T51A MazF mutants in *E. coli* Rosetta (DE3) pLysS results in normal growth after IPTG induction, whereas K79A and R86A MazF mutants and WT MazF inhibit cell growth [Fig. 6 ▸(*d*)]. This demonstrates that Arg28 and Thr51 play a critical role in *K. pneumoniae* MazF ribonuclease activity.

In general, MazF homologs adopt a PemK-like fold that cleaves single-stranded mRNA transcripts (Hoffer *et al.*, 2017 ▸). Several conserved residues of *K. pneumoniae* MazF could be identified by comparison with its structural homologs. Among them, Arg28 and Thr51 are the key residues involved in RNA catalysis. As a result, the mechanism of action of *K. pneumoniae* MazF is different from that of other ribo­nucleases, such as RNase H and VapC toxin, which are magnesium-dependent ribonucleases. For RNase H, a water molecule coordinated by Mg^2+^ begins a nucleophilic attack on a scissile phosphate of the RNA strand, forming a pentacovalent intermediate. After that, another water molecule reprotonates the 3′-OH group, resulting in the formation of 5′ phosphate and 3′-OH groups (Yang *et al.*, 2006 ▸). In contrast, for *K. pneumoniae* MazF, following the buildup of the negatively charged phosphate on the cleavage site stabilized by Thr51, the product, namely, 2′,3′-cyclic phosphate, is formed via a Grotthuss-like mechanism (Zorzini *et al.*, 2016 ▸). In this case, the conserved Arg28 acts as a general base and general acid by transferring a proton to the cleavage site. However, the mutation on Thr51 of *K. pneumoniae* MazF indeed affected its ribonuclease activity, while the alanine mutant of the corresponding threonine showed only a small decrease in ribonuclease activity in some other MazFs (Ahn *et al.*, 2017 ▸; Hoffer *et al.*, 2017 ▸). This is because two additional threonines existing in the active site of these other MazFs compensate for the role of the conserved threonine, while the sole threonine existing in the active site of *K. pneumoniae* MazF contributes to catalysis.

## 4. Conclusions   

Based on the high-resolution crystal structure of *K. pneumoniae* MazEF, we were able to both analyze the tertiary structure of the full-length MazF toxin and clearly identify the binding interface of MazEF. Loop β1–β2 was found to play the most important role in MazF, as it functions as a gate to modulate an open to closed state. When MazE binds with MazF, it not only displaces the β1–β2 linker to induce an open state but also occupies Site A of the MazF homodimer, resulting in blockage of the downstream region of the RNA-binding site. In addition, Arg28 and Thr51, which lie in the RBP, are the key residues for the catalytic reaction. The results for the binding interface provide valuable information for designing peptides or small molecules to artificially disrupt the MazEF complex. If we could design substances that display high affinity for the TA interface pocket, free MazF toxin that possesses a naked site A and an active site will be produced, resulting in ribonuclease activity. In conclusion, the tertiary structural information reported here will contribute to the exploration of antimicrobial candidates to treat drug-resistant *K. pneumoniae*.

## Supplementary Material

PDB reference: *Klebsiella pneumoniae* MazEF, 7bye

## Figures and Tables

**Table 1:** Data-collection and refinement statistics for our crystal structure Values in parentheses are for the highest-resolution shell.

Data-collection details	
X-ray source	11C beamline of PLS, Republic of Korea
X-ray wavelength (Å)	0.9794
Space group	*P*1
Unit-cell parameters	
*a*, *b*, *c* (Å)	41.558, 45.501, 69.952
α, β, γ (°)	102.06, 93.60, 116.36
Resolution range (Å)	50.00–2.30
Molecules per asymmetric unit	2 MazEF heterotrimers
Observed reflections (>1σ)	56085
Unique reflections	17681
〈*I*/σ(*I*)〉	18.9 (7.7)
Completeness (%)	89.5 (91.2)
Multiplicity†	3.2 (3.2)
*R*_p.i.m._ (%)‡	6.7 (11.1)
CC_1/2_, CC	(0.946, 0.986)
Refinement statistics	
*R*_work_ (%)§	19.8
*R*_free_ (%)¶	23.2
No. of atoms/r.m.s.	3644/3.04
R.m.s.d. from ideal geometry††	
Bond distance (Å)	0.006
Bond angle (°)	1.069
Ramachandran statistics	
Most favored regions (%)	97.30
Additional allowed regions (%)	2.70
Residues in disallowed regions (%)	0.00
PDB accession code	7bye

† *N*_obs_/*N*_unique_.

‡ *R*_p.i.m._ = ∑_*h*_[1/(*n*_*h*_ − 1)]^1/2^∑_*i*_|〈*I*_*h*_〉 − *I*_*h*, *i*_|/(∑_*h*_∑_*i*_ *I*_*h*, *i*_).

§ *R*_work_ = ∑*_hkl_* ||*F*_obs_| − *k* |*F*_calc_||/(∑*_hkl_* |*F*_obs_|).

¶ *R*_free_ was calculated in the same way as *R*_work_, but with 5% of the reflections excluded from the refinement.

†† R.m.s.d. was calculated with *REFMAC*.
